# Adrenal suppression due to an interaction between ritonavir and injected triamcinolone: a case report

**DOI:** 10.1186/1742-6405-6-10

**Published:** 2009-06-08

**Authors:** Kathryn Dort, Shetal Padia, Brian Wispelwey, Christopher C Moore

**Affiliations:** 1 Department of Medicine, Division of Infectious Diseases and International Health, University of Virginia School of Medicine, Charlottesville, Virginia, USA; 2 Department of Medicine, Division of Endocrinology, University of Virginia, School of Medicine, Charlottesville, Virginia, USA

## Abstract

Two HIV-1 infected patients developed signs and symptoms consistent with adrenal suppression after being exposed to intra-articular triamcinolone acetate while also receiving ritonavir as part of their highly active antiretroviral therapy. Laboratory evaluation confirmed secondary adrenal suppression in both cases. Both patients recovered without the need for chronic replacement steroids. Adrenal suppression has been described as an adverse outcome in patients treated with fluticasone and concomitant ritonavir. In the reported cases, the adrenal suppression likely developed as a result of increased systemic concentrations of triamcinolone due to an inhibition of cytochrome p450 3A4 metabolism. Practitioners of HIV medicine should be aware of the potential negative interaction of injected triamcinolone and ritonavir.

## Introduction

Ritonavir reduces the metabolism of systemic steroids including inhaled fluticasone which may lead to clinical Cushing's syndrome and secondary adrenal insufficiency [1-3]. Therefore, the decision to use inhaled or systemic steroids in conjunction with ritonavir should be made with caution. Despite occasional reports of Cushing's syndrome occurring with injected triamcinolone even in the absence of cytochrome p450 3A4 inhibitors, it is not clear that the same caution should be exercised when considering local steroid injections in the setting of ritonavir therapy [4,5]. Here we present two cases of adrenal suppression which occurred after intra-articular injections of triamcinolone in HIV-1 infected persons receiving ritonavir as part of their antiretroviral regimen.

## Case report

### Case 1

A 41 year-old HIV-1 infected man presented to our clinic with concerns about non-healing abdominal bruising that he related to a motor vehicle collision that occurred approximately 6 weeks earlier. He noted weight gain without a change in his appetite or food intake. He complained of a pruritic rash on his upper chest and arms which he had noticed for approximately one month. His HIV-1 infection was treated daily with the fixed-dose combination of 200 mg emtricitabine and 300 mg tenofovir as well as 100 mg ritonavir and 300 mg atazanavir. He had been on a ritonavir boosted protease inhibitor (PI) regimen for four years. His CD4+ T lymphocyte concentration was 842/μL and his viral load was undetectable (level of detection <50 copies/mL-Roche v 1.5). He had been vaccinated against hepatitis A and B and uninfected by hepatitis C. He denied taking any inhaled or oral steroids, but due to chronic low back pain he had received two transforaminal epidural injections of 80 mg triamcinolone acetonide at an outside facility approximately 3 and 2 months prior to presentation.

**Figure 1:**
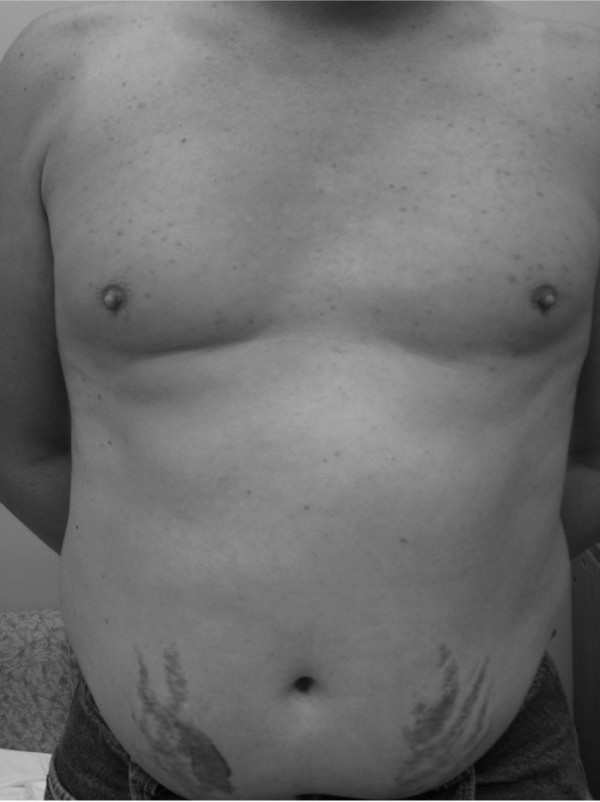
**Patient 1 with stigmata of Cushing's syndrome including acneiform rash, truncal obesity, and abdominal striae**.

His blood pressure was 144/88 mmHg and his pulse was 91 beats per minute which were elevated from his baseline of approximately 100/75 mmHg and pulse of 80 beats per minute. His weight had increased by approximately 15 kg from his prior clinic visit 4 months prior. He had notable truncal weight gain and new Cushingoid facies. He had prominent 1 cm wide purple striae on the anterior abdomen with scattered striae on the flanks bilaterally and acneiform lesions on the chest, shoulders, back and upper arms (Figure 1).

A mid-morning cortisol concentration was 13.80 nmol/L and his adrenocorticotropic hormone (ACTH) concentration was <0.22 pmol/L (normal range 1.98–11.44 pmol/L). His thyroid-stimulating hormone concentration, electrolytes and renal function were all within normal limits. A synthetic glucocorticoid steroid blood screen revealed a triamcinolone acetonide concentration of 98.9 mmol/L (expected cutoff 6.9 mmol/L). He was counseled on the symptoms of adrenal crisis but continued his antiretroviral regimen without steroid replacement.

One month after his initial presentation to clinic, his symptoms had improved, he was normotensive, and his weight was reduced by 4 kgs. Two months later due to complaints of left hip pain an anterioposterior roenterogram of the pelvis and left hip was obtained and revealed a large area of avascular necrosis within the left femoral head with significant lateral cortical lucency. Four months later, a mid-morning cortisol was 33.10 nmol/L, his ACTH concentration was 1.32 pmol/L, CD4+T lymphocyte concentration was 693/μL and his viral load remained undetectable. At his 6 month follow up visit, his afternoon random cortisol and ACTH values had returned to normal range (427.65 nmol/L and 5.72 pmol/L, respectively).

### Case 2

A 42-year-old HIV-1 infected woman presented to our clinic with complaints of weight redistribution around the neck and upper thighs, weakness, heat intolerance, blurry vision, heart palpitations, fatigue, hyperexcitability, insomnia, and increased appetite for approximately 20 days. Her HIV-1 infection was treated daily with the fixed dose combination of 200 mg emtricitabine and 300 mg tenofovir as well as daily 100 mg ritonavir and 300 mg atazanavir. Her most recent CD4+ T lymphocyte concentration was 693/μL and her viral load was undetectable. She had been vaccinated against hepatitis A and B and was not infected with hepatitis C.

Upon presentation to our clinic she was found to have a blood pressure of 152/100 mmHg which was elevated from her baseline of 100/58 mmHg. Thyroid function studies, electrolytes and renal function were all within normal limits. Further evaluation revealed a mid-morning cortisol concentration of 55.18 nmol/L which increased after 0.25 mg cosyntropin injection to 386.26 nmol/L at 60 minutes (normal response at 60 minutes is >469.03 nmol/L). Her morning ACTH concentration was < 0.22 pmol/L.

She denied using inhaled, oral or topical steroids. She had not been prescribed medroxyprogesterone or megestrol acetate. Due to a right shoulder impingement, she did receive an injection of 40 mg triamcinolone acetonide in her right subacromial space at an outside facility two weeks prior to her symptom onset. Six months prior to that she received a transforaminal epidural injection of betamethasone acetate as treatment for cervical spondylosis without complications while receiving the fixed-dose combination of lamivudine and zidovudine plus efavirenz. Initially her adrenal suppression was treated with a short burst of hydrocortisone (30 mg daily) to prevent potential adrenal crisis but this was discontinued after three days. Two months later the patient was asymptomatic and her random afternoon cortisol was 110.36 nmol/L, ACTH 1.76 pmol/L, CD4+ T lymphocytes 444/μLand viral load remained undetectable.

## Discussion

Cushing's syndrome is known to occur with high doses of exogenous steroids, but has rarely been associated with triamcinolone injections [4-6]. Our patients' symptoms occurred approximately two weeks after intra-articular injection of triamcinolone acetonide while they were also receiving the fixed-dose combination of emtricitabine and tenofovir plus ritonavir and atazanavir. They had no history of concomitant inhaled, intranasal or topical steroids. Therefore, their adrenal suppression likely represents a drug interaction between ritonavir, a known inhibitor of steroid metabolism, and intra-articular injection with triamcinolone acetonide.

The ability of ritonavir to inhibit cytochrome P450 3A4 (CYP 3A4) is exploited to increase the bioavailbility of other PIs and increase their dosing intervals [7,8]. However, ritonavir increases the concentration of exogenous steroids through the same mechanism. One pharmacokinetic study revealed a 28% increase in prednisolone exposure when ritonavir was co-administered with oral prednisolone, the active metabolite of triamcinolone. This was thought to occur due to the inhibition of the CYP 3A4 system, the primary method of metabolism of prednisolone [3]. Prednisolone is also known to have an increased area under the plasma concentration versus time curve and decreased oral clearance when combined with ritonavir [9]. These findings are similar to the interaction of other CYP3A4 inhibitors, e.g. itraconazole, with prednisolone. This rapid, increased exposure to exogenous glucocorticoids may lead to clinical Cushing's syndrome and suppression of the hypothalamic-pituitary-adrenal (HPA) axis which may last from nine months to a year [10,11].

In a pharmacokinetic study of intra-articular administration of triamcinolone acetonide endogenous hydrocortisone suppression correlated with exogenous steroid concentrations and triamcinolone was fully absorbed within two to three weeks[12]. Therefore, when our patients presented to our clinic several weeks after their intra-articular injections we would not have expected them to have such profound HPA axis suppression from triamcinolone alone, or in case 2 from a betamethasone injection 6 months prior. On the contrary, our cases corroborate the concern raised by two other recently published reports of adrenal insufficiency following administration of intra-articular injections of triamcinolone acetonide 40 mg in patients infected with HIV-1 receiving a ritonavir boosted PI regimen [13,14].

Once iatrogenic adrenal suppression is suspected, a random, preferably morning, serum cortisol and ACTH should be obtained. An ACTH (cosyntropin) stimulation test can confirm adrenal axis suppression caused by exogenous glucocorticoids. In Case 1, the synthetic glucocorticoid steroid screen confirmed that the prior triamcinolone acetonide injection was the source of exogenous steroids and presumably adrenal suppression. Usually careful history taking will provide the source of exogenous steroids, but this screening test may be useful in cases where history is lacking but clinical suspicion is high.

In cases of adrenal suppression due to exogenous glucocorticoid administration, physiological replacement with hydrocortisone may not be necessary and chronic use of supraphysiological doses of corticosteroids should be avoided [11]. Corticosteroids, usually hydrocortisone, may be necessary in the acute setting of adrenal insufficiency which can be apparent at diagnosis and with subsequent periods of stress (e.g. trauma, surgery or severe illness) [11]. Additionally, when further steroids are required it may be necessary to substitute non-nucleoside reverse transcriptase inhibitors or newer agents such as integrase inhibitors or CCR5 inhibitors for ritonavir boosted PIs. It is also important to avoid other CYP 3A4 inhibitory drugs such as itraconazole which may also increase the concentration of circulating corticosteroids. Avoidance of chronic corticosteroid replacement in both of these patients likely allowed speedier recovery of their HPA axes without the need to switch from their ritonavir boosted PI regimens from which they both had good virologic response.

A high index of suspicion for adrenal suppression is required when considering protean symptoms of a ritonavir treated HIV-1 infected patient who has recently received corticosteroids. As with our patients, careful history taking and physical examination are required to make the diagnosis and reveal the source of glucocorticoid exposure. The diagnosis may be obscured by a prior history of lipodystrophy which has similar clinical findings to those of Cushing's syndrome. However, a diagnosis is crucial to avoid the myriad complications of adrenal suppression and excess exogenous glucocorticoids which may include neuropsychological changes, hypertension, diabetes, osteoporosis and necrosis, and immune deficiency, among others.

## Conclusion

As the HIV-1 infected population with access to antiretroviral therapy ages they are likely to encounter diseases with a predilection for the elderly such as degenerative joint disease and osteoarthritis. Due to the frequent use of ritonavir in antiretroviral regimens and the common practice of intra-articular injection of steroids for rheumatic diseases, more research is needed to evaluate the interaction of injected steroids and ritonavir We advocate that any use of steroid supplementation, including intra-articular injection, should be used with caution in the setting of concurrent use of ritonavir.

## Consent

Written informed consent was obtained from the patients for publication of their case report and the accompanying image. A copy of the written consent is available for review by the Editor-in-Chief of this journal.

## Competing interests

The authors declare that they have no competing interests.

## Authors' contributions

All authors participated in the drafting of the manuscript. All authors read and approved the final manuscript.
